# The New York Institute of Stomatology

**Published:** 1903-05

**Authors:** 

**Affiliations:** The New York Institute of Stomatology


### Reports of Society Meetings.

### THE NEW YORK INSTITUTE OF STOMATOLOGY.

A MEETING of the Institute was held at the "Chelsea," No. 222 West Twenty-third Street, New York, on Tuesday evening, February 3, 1903, the President, Dr. J. Morgan Howe, in the chair.

The minutes of the last meeting were read and approved.

COMMUNICATIONS ON THEORY AND PRACTICE.

Dr. Gillett presented some paper points, for use in drying pulp-canals, which had recently been put upon the market, at his suggestion, by Johnson & Johnson. The points were put up in convenient form and thoroughly sterilized. He stated his belief that at least half of the difficulties in the past with pulpless teeth were due to the failure of the operators to observe aseptic precautions.

Dr. E. A. Bogue presented a model, taken ten years ago, of an appliance made by Dr. Bing, and which had been already in the mouth ten years when the impression was taken. Dr. Bogue called attention to it as containing the gist of an appliance that Dr. Carmichael is now bringing before the profession.

Dr. Herbert L. Wheeler then read his paper on "Porcelain."

(For Dr. Wheeler's paper, see page 329.)

DISCUSSION.

Dr. W. A. Capon, of Philadelphia, wished to thank the essayist for giving the Institute a paper a little out of the routine on porcelain. Many practical points had been brought out. He objected to the term "faddism" in this connection, and thought that porcelain inlays had reached a point of realism and substantiality, from a conservative stand-point. He could only speak upon the subject from a practical stand-point.

The first subject brought to his attention by the essayist was the relative strength of English and American teeth. The difference was not so great as one would infer from listening to the paper. The English teeth were strong, although they fused at a lower temperature.

Dr. Capon advocated the high-fusing inlay material, although he admitted the superiority of gold as a matrix in certain places. However, he found very little difficulty in getting a matrix with platinum which adapted itself to the walls of the cavity, but it was seldom possible to get an impression of the floor of the cavity also. He did not think the point made of burning out the color amounted to anything, as with a little practice that could be avoided in any case. He had seen the shades burned out of the high-fusing bodies also. There was not so much difference in the shrinkage either. The Jenkins body was a terrific shrinker. The Ash body was also a great shrinker, both high and low. The S. S. White, that fused at

2300°, was almost as great a shrinker. The Close, at 2300°, shrunk less than any. Brewster's shrunk considerably.

Dr. Capon did not know why the term "inlay" came to be applied to these fillings so promiscuously. Until Dr. Head, of Philadelphia, had used the term, he had always spoken of them as porcelain fillings. He thought the terms should be divided, and the word "inlay" be applied to such fillings as occurred in the labial margins. But a corner or an approximal contour should be spoken of as a "porcelain section."

So far as the different porcelain bodies were concerned, he used Close's to-day in connection with Whiteley's and the S. S. White.

Dr. R. T. Moffatt, of Boston, thought that the question of true porcelain was one that was not settled by the essayist's definition. It might be interesting to look at some of the formulæ of porcelain teeth. These formulæ generally were composed of a base that represented the dentine of the natural tooth and an enamel that represented the natural enamel. Artificial enamel was composed mostly of felspar and coloring frit, and the body of silica, felspar, clay, and the coloring. The following formula made a very excellent body: Spar, 12 pennyweights; silica, 8 pennyweights; clay, 20 grammes. The shrinkage of this body (about one-eighth) was not very great, which was very essential in carving teeth.

The essayist spoke of porosity. He did not state that porosity could be due to faulty manipulation. The body was mixed with water or alcohol, as the case might be, and if baked when it is pretty wet, porosity might result, but if put in quite dry and packed hard, the same body would be found to be very dense.

On account of its lack of strength the essayist seems to think the use of porcelain as a filling quite limited. In this there were several things to consider. Porcelain would stand a considerable crushing stress, steadily applied, whereas a sudden impact would fracture it.

Regarding durability, all true porcelain was indestructible in the fluids of the mouth. There was no doubt about that. The essayist stated that glass might be dissolved by the fluids of the mouth after a certain length of time. Dr. Moffatt quite agreed with that. The surface of these inlays turned black, not on account of the absorption of foreign particles, but on account of the presence of oxide of lead, which was the principal base in flint and crystal glass. That might indicate that these modern enamels were not glass but porcelain.

Dr. Capon spoke of burning out colors. In the tooth-carving bodies it was almost impossible to burn out the colors. The principal color in yellow teeth was oxide of titanium, and it took a great degree of heat to even bring that color out. Many times the shade depended upon the size, especially the depth, of the inlay.

The essayist did not give the formula of Dr. Jenkins's body. Dr. Wilson, of Boston, with whom Dr. Moffatt is associated, at the time Dr. Jenkins brought out his material got interested in the subject. Dr. Wilson's idea was that Dr. Jenkins's enamels were practically like gum frit, with the exception of a change of color.

Dr. Capon spoke of burnishing a matrix to the bottom of a cavity. Dr. Moffatt could not see the necessity of always obtaining an exact impression of the bottom of the cavity, provided the walls and margins were perfect. Dr. Capon also stated that in some porcelain fillings it was never possible to tell what the color would be until they were finished. This was one argument in favor of the platinum matrix, as it did not require investing and could be inserted in the cavity from time to time during the process of making to compare the shade. Of the inlay bodies, he considered the S. S. White and the Brewster the best for high fusing and the Jenkins for low fusing. Dr. Moffatt did not consider, in making porcelain fillings, that the question of shrinkage amounted to so much, as the matrix could be refilled at each bake until the required contour was obtained, but it was very important in tooth carving, as the proper allowance for shrinkage had to be made at the beginning.

Dr. E. A. Bogue thought it was not necessary to speak words of commendation of Dr. Wheeler's paper. Dr. Moffatt had alluded to the fact that glass was unfit for fillings on account of the fact that glass contained lead. However, glass could be made without any lead in its composition. As there had been a great deal of allusion to Dr. Jenkins's low-fusing inlay material, Dr. Bogue desired to bring up some rather curious remarks regarding it.

Regarding platinum as a matrix, he thought the trouble was that we did not get it pure. It generally came alloyed with some other metal, as iridium, which made it very much harder. When pure there was very little difference between it and gold.

Dr. Bogue stated that Dr. W. H. Rollins, of Boston, began practice in 1873 or 1874, and that Dr. Rollins had written him that he had begun work on porcelain fillings at once and that by

1879 he was using them with success. In June, 1880, Dr. Rollins read a paper upon this subject before the Boston Society for the Advancement of Oral Science, in which paper he described every method he had thought of for making metal moulds for porcelain fillings. Later he published a paper in the *Archives of Dentistry* for January, 1885, in which paper is a cut of the "revelation bur," which he writes he had sent to White's long before and asked them to make and which the White Company afterwards patented, giving him no credit. This paper also contained formulæ for dental enamels and porcelains required for use in fillings. Dr. Rollins worked long and earnestly to find the cause of discoloration in porcelain fillings, and, according to a translation of Dr. Bruck's paper published in the *Items of Interest* for September, 1902, a new development of porcelain fillings began from the work of Dr. Rollins. Pieces of artificial teeth fused into a platinum impression of the outer borders of the cavity had been ascribed to Dr. Land, of Detroit, as early as 1870, but Dr. Bogue found no record of his making complete inlays to fit the cavity, nor did he find anywhere else in the history of porcelain fillings any such attempt at accuracy as was shown by Dr. Rollins's work.

### DR. ROLLINS'S LETTER.

BOSTON, February 2, 1903.

DEAR DR. BOGUE,—I cannot refer you to a paper by Dr. Jenkins in which he claims to have been the first to use gold- or platinum-foil matrices for moulds in which to fuse ceramic material for the production of fillings for teeth, as I am not very familiar with his writings. But I have always supposed he made this claim, as I have never seen a paper in which he credited me with the invention, and if he does not claim this, what is the Jenkins system of filling teeth? It becomes not a system, but a secret material, which he makes and sells to dentists, and thereby he comes into the same class as Ash, Brewster, White, and other manufacturers, who do the same thing. Moreover, he translated a paper by Bruck and had it published in the *Items of Interest* as late as 1892, in which he (Bruck) purports to give a history of ceramic fillings, and to which history the translator makes no corrections. In this historical survey the date of my paper selected as fixing the date of my first contribution is given five years too late; that is, the paper selected is one published two years after my first one; and he fails to state that the paper he puts on record as the first is but an abstract of a paper read before a dental society *five* years earlier. Moreover, he ignores my later paper, in which I stated that my first method was to take the foil matrix directly from the cavity, and he then goes on to say that the method described in my paper *was too com-*

*plicated to be of use.* Secondly, he states in italics that in 1870 Land made the experiment of fusing pieces of artificial teeth in a platinum impression of the outer borders of the cavity. He does not tell where to look for Land's paper. Then he said, "Thus in 1890 Professor Sachs recommended taking the impression with Williams's gold-foil and platinum-foil No. 60 instead of Stent's Composition, and by means of this mould, *obtained direct from the cavity,* secured superior exactness of the margins." As this paper reads, therefore, Dr. Jenkins having made no corrections in it, Dr. Land made impressions of the edges and Sachs made impressions of the cavity in metal foil. Now, long before this paper was published one of Land's friends, whom I might expect to know, claimed that Land was the discoverer of moulded ceramic fillings, and gave the date of the discovery as 1887 (see page 721 of *Items of Interest* for 1899), and he dates the era of porcelain from that time and that invention. Again, on page 727 Dr. Capon gives Dr. Land the credit and fixes the date at 1886. Now, it seems to me that if Land had published a paper on foil matrices in 1870, as stated in Jenkins's translation, these men should have known of it, as they were evidently Land's friends and were trying to prove that the so-called Jenkins method was not invented by him, but by Land. I therefore infer from this that they and others, to whom Dr. Jenkins showed his methods, thought that he was claiming metal-foil matrices as his invention. Every one I have talked with thought so to. If Dr. Jenkins at that time was not claiming to be the originator of a new method of filling, but only appearing as a manufacturer of a filling-material, I cannot see why there should have been any discussion about the originator of the method. The discussion would have been confined to the relative merits of the different ceramic materials made by the different manufacturers, among whom was Dr. Jenkins.

I shall be glad to have you use any facts I have given you in any way which you think is likely to make the history of ceramic filling clear, and if at any time I find that some one before my day invented the foil matrix, which is to all intents and purposes ceramic filling, I shall be very glad to withdraw my claim.

Yours sincerely,

WILLIAM ROLLINS.

Dr. Bogue had written Dr. Rollins quite a long letter regarding the work of Dr. Herbst, a German. Dr. Herbst spoke no English, and had worked at glass fillings with platinum and gold matrices for a long time. Through that work he introduced the matrix system of fillings into Germany.

Dr. N. S. Jenkins, who has lived in Germany for thirty or forty years, and who had probably never seen nor heard of the *Boston Medical and Surgical Journal,* saw some good in the system introduced into Germany by Herbst and undertook to improve upon it. In that effort he had spent most of his time in the past four or five years, denying himself the lesser pleasures of social intercourse and enlisting in his work, through his royal friend and patron, the managers and workers of the porcelain factory at Dresden. He had expended somewhere near ten thousand dollars in the experiments that are still going on. He has travelled largely in America, France, Germany, Sweden, and Denmark to introduce this system and to present it clinically, gratuitously teaching the technique to any one willing to learn. This coming spring he was going to Spain to attend the meeting of the International Medical Congress, where he is slated for a paper on porcelain fillings. Dr. Bogue mentioned all this because he had spent a week with Dr. Jenkins last summer and had seen the serious work that he was doing.

Dr. Jenkins claims that by baking his porcelain three times lightly and a fourth time thoroughly, he succeeds in getting a substance so dense and hard that it will scratch glass; so strong that it is practically impossible to break it when it is in position; so perfectly adapted, if the matrix is perfect, that nothing further could be desired, and, finally, if the heat be regulated as Dr. Jenkins now claimed to be able to do, the color would be absolutely exact also. The way Dr. Jenkins regulated his heat was to use a gas blow-pipe with a shut-off attachment that had been tested previously according to the gas pressure until the proper degree of heat had been ascertained. When the first three bakes were made the heat was brought barely up to the fusing-point and then stopped, while at the last baking the heat was brought to the fusing-point and kept there, not increased, the same degree of heat being maintained, until complete fusion took place. This, it was claimed by Dr. Jenkins, made a homogeneous mass and avoided bubbling, which had been such an obstacle to perfection and which the essayist of the evening referred to as a chemical change which had taken place at high temperatures only. Dr. Bogue thought that in this particular the essayist agreed theoretically with Dr. Jenkins's conclusions, however much he might differ from him on general grounds.

One of the main grounds for failure in the use of porcelain fillings was a ground that existed for the failure of all fillings, but was not generally recognized,—viz., the extension of disintegration to a point considerably beyond the border of the cavity, after it is supposed to be fully excavated. Dr. Wedelstaedt seemed to have recognized the condition, but he put it differently and called it "extension for prevention." Only last week Dr. Bogue had occasion to insert two porcelain fillings at the cervical margin, labial side, of two upper cuspids. This was a position of all others, the upper incisors excepted, where we should expect the surfaces to be free from deleterious deposits; yet with the rubber dam in place for an hour, so that the surfaces became well dried, the difference in color between the lower margins of both cavities and the bulk of the teeth was perceptible for at least one-sixteenth of an inch from the margins of the cavities. It would have been manifestly improper to have extended these cavities in order to prevent decay, but he had shown the traces of the destroyer to his patient and had encouraged her to continue her cleanly habits, using lime water as a detergent as well as an antacid. If traces of disintegration could be discovered in such localities, what might be expected in cases of approximal decay far back in the mouth, where often the light could not be made to penetrate and where direct vision was impossible. Another objection that occurred to Dr. Bogue was where the inlays, after the lapse of years, struck against occluding surfaces.

Dr. C. F. Allan said that while the essayist's paper presented the subject in a light different from anything before written, and had brought out many details of the greatest interest in relation to physical structure and composition, he was afraid that when we come to the practical questions as to when and where to use porcelain, we would be as much in the dark as ever.

Dr. Allan thought that the range for the use of this material was very much greater than had even been suggested by any of the speakers this evening. The statement that its use should be bounded solely by considerations of æsthetics relegates the material to a minor position such as is not at all warranted by its merits.

Just to make one exception to his narrow construction of the use of porcelain, he would like to call attention to its great value for filling and restoration of contour in cases of badly broken-down bicuspid and molar teeth, especially of the upper jaw.

When we think of the serious undertaking it is to fill such teeth with gold, contouring and finishing to that degree of perfection absolutely necessary for good results, then to know that we can do all that contouring in the furnace, it certainly does not seem wise or reasonable to limit the use of porcelain in this narrow way. Such cases are thoroughly practical, have, as a rule, no difficulties that are at all insuperable, and compare favorably for permanence with any other way. Comfort, permanence, and æsthetics are all subserved.

As affecting the great advantages of gold in making matrices, it is being continually stated that platinum has a great advantage in that it can be returned to the cavity after the first and subsequent bakings to be burnished to a perfect fit. To the users of Jenkins bodies this is not at all a merit, as he expects to make a perfect matrix and from a perfect matrix will have perfect work.

Until more scientific, careful, and detailed experiments are made as to the comparative merits of high- and low-fusing bodies it is useless to criticise, but the users of Jenkins bodies have so far no reason to doubt the great merits of this material.

Dr. Gillett complimented the essayist. He had presented to the profession a line of thought never before worked out. Dr. Gillett thought it time we stopped discussing the advantages of one form of porcelain over another and recognized the fact that they were both adapted for certain cases. In selected places he was able to use Dr. Jenkins's material more rapidly and just as satisfactorily as he could the high-fusing body. Personally he was no more interested in the discussion as to which was the better, the high- or the low-fusing body, than he was in the discussion as to the relative superiority of gold and amalgam. Although they were not used in the same place, they both had their uses. He was satisfied that the Jenkins material was sufficiently durable when properly manipulated, and found it especially adapted for labial and approximal cavities in incisors and cuspids. The essayist doubted if a filling could be made with a concavity from the Jenkins material. If he would try it he would find that it could be done. Dr. Gillett thought that the shadow problem was more troublesome in the low- than in the high-fusing material that contained more of the insoluble ingredients.

Dr. Wheeler, in closing the discussion, said that, regarding shrinkage, if there were a large amount of kaolin present in the formula the shrinkage would be great, and that the opposite would be true if there were a large amount of silica.

In regard to glass, there was present in flint glass about thirty-three per cent. oxide of lead. However, there were several kinds that did not contain oxide of lead at all. It did not necessarily form a base in the making of glass.

What Dr. Moffatt had said about bubbling was true. Moisture did have a great deal to do with it. He had stated in his paper that chemical change contributed to bubbling. He did not claim that it was the sole cause. Regarding insolubility, he did not dispute the claim that it was possible to produce a low-fusing body that would be practically insoluble. That remained to be seen.

In regard to the oxide of titanium, he learned from his sources of information that it did burn away as a coloring-matter.

As regards Dr. Rollins being the first to use the metal matrix for porcelain fillings, Dr. Wheeler had seen a recorded description of the process by him as early as 1885, but he had been unable to find any recorded statement of Dr. Land's that would give him the credit for having first brought it before the profession.

Regarding the fusing-points, these were not from his own experiments, but were given to him by Mr. Hammond. He doubted the accuracy of some of them.

Regarding the question of gold *versus* platinum as a matrix, he had seen very fine porcelain fillings made in both forms of matrix. He thought it was a question of workmanship. He did not think one could take either Dr. Capon or Dr. Gillett as a fair example of what the average individual would do with these materials.

Upon motion, a vote of thanks was extended to both Dr. Capon and Dr. Moffatt for their kindness in coming on from distant cities to discuss the paper.

Dr. G. S. Allan presented to the Institute a request from Dr. Louis Jack that he have the privilege of soliciting from active or associate members of the Institute original papers and contributions. Dr. Allan stated that, as the request seemed so reasonable, he would move that it be granted. Motion carried.

Adjourned.

FRED. L. BOGUE, M.D., D.D.S.,
*Editor The New York Institute of Stomatology.*

